# Early Treatment Response in Non-Small Cell Lung Cancer Patients Using Diffusion-Weighted Imaging and Functional Diffusion Maps – A Feasibility Study

**DOI:** 10.1371/journal.pone.0108052

**Published:** 2014-10-07

**Authors:** Carolin Reischauer, Johannes Malte Froehlich, Miklos Pless, Christoph Andreas Binkert, Dow-Mu Koh, Andreas Gutzeit

**Affiliations:** 1 Institute of Radiology and Nuclear Medicine, Clinical Research Unit, Hirslanden Hospital St. Anna, Lucerne, Switzerland; 2 Department of Radiology, Cantonal Hospital Winterthur, Winterthur, Switzerland; 3 Department of Radiology, Paracelsus Medical University Salzburg, Salzburg, Austria; 4 Department of Oncology, Cantonal Hospital Winterthur, Winterthur, Switzerland; 5 Academic Department of Radiology, Royal Marsden NHS Foundation Trust, Sutton, Surrey, United Kingdom; 6 CR-UK and EPSRC Cancer Imaging Centre, Institute of Cancer Research, Sutton, Surrey, United Kingdom; West German Cancer Center, Germany

## Abstract

**Objective:**

The aim of this study was to prospectively evaluate the feasibility of monitoring treatment response to chemotherapy in patients with non-small cell lung carcinoma using functional diffusion maps (fDMs).

**Materials and Methods:**

This study was approved by the Cantonal Research Ethics Committee and informed written consent was obtained from all patients. Nine patients (mean age = 66 years; range = 53–76 years, 5 females, 4 males) with overall 13 lesions were included. Imaging was performed within two weeks before initiation of chemotherapy and at one, two, and six weeks after initiation of chemotherapy. Imaging included a respiratory-triggered diffusion-weighted sequence including three b-factors (100, 600, and 800 s/mm^2^). Treatment response was defined by change in tumor diameter on computed tomography (CT) after two cycles of chemotherapy. Changes in the apparent diffusion coefficient (ADC) on a per-lesion basis and the percentages of voxel with significantly increased or decreased ADCs on fDMs were analyzed using repeated measures analysis of variance (ANOVA). Changes in tumor size were used as covariate to examine the ability of ADCs and fDM parameters to predict treatment response.

**Results:**

Repeated measures ANOVA revealed that the percentage of voxels with increased ADCs on fDMs (p = 0.002) as well as the mean ADC increase (p = 0.011) were significantly higher in good responders with a large reduction in tumor size on CT.

**Conclusion:**

Our results indicate that the percentage of voxels with significantly increased ADCs on fDMs seems to be a promising biomarker for early prediction of treatment response in patients with non-small cell lung carcinoma. Contrary to averaged values, this approach allows the spatial heterogeneity of treatment response to be resolved.

## Introduction

Response to anticancer drugs of non-small cell lung cancer is usually evaluated as tumor shrinkage on computed tomography (CT) after two cycles of chemotherapy in agreement with the response evaluation criteria in solid tumors (RECIST) [1]. Novel chemotherapies as well as new targeted therapies are being progressively introduced, therefore new biomarkers that permit early treatment monitoring and the prediction of treatment response are warranted so that treatment can be more rapidly adapted, avoiding unnecessary adverse effects from ineffective treatment and rendering anticancer therapies more cost efficient.

Diffusion-weighted imaging (DWI) is a promising tool for evaluating treatment response to anticancer therapy at an earlier stage than tumor size measurement, since cellular death and vascular changes precede changes in lesion size [2]. Using DWI, the apparent diffusion coefficient (ADC) can be calculated which has been shown to be a useful quantitative response biomarker to anticancer drugs in brain tumors [3], breast cancer [4]–[6], head and neck tumors [7], [8], cervical cancer [9], liver cancer [10], rectal cancer [11], soft-tissue sarcomas [12], bone metastases [13], [14], and non-small cell lung carcinoma [15].

More recently, the functional diffusion map (DM) has been investigated as a method of voxelwise ADC analysis that is potentially more sensitive in detecting treatment response than ADCs averaged over entire lesions [16], [17]. The fDM characterizes and quantifies heterogeneity of treatment response by segmenting the tumor on a voxelwise basis into three distinct regions with significantly increased (red voxels), significantly decreased (blue voxels) and unchanged ADCs (green voxels) under therapy. Using this method, studies have up until now focused largely on the investigation of brain tumors [16]–[19]. The translation to body regions prone to motion is challenging due to the requirement of precise coregistration of the pre- and post-treatment ADC maps as a mandatory preprocessing step for calculating fDMs. Hence, fDM analysis of lesions in the lung is particularly challenging due to respiratory motion and susceptibility-related artifacts caused by tissue-air interfaces.

Thus, the aim of the present pilot study was to prospectively evaluate the feasibility of monitoring and predicting treatment response in patients with non-small cell lung cancer using fDMs compared with reduction in tumor size on CT after two cycles of therapy as the reference standard.

## Materials and Methods

### Study Population

This study was approved by the Cantonal Research Ethics Committee and informed written consent was obtained from all patients in our prospective clinical study conducted between August 2010 and August 2012. Nine patients (mean age = 66 years; range = 53–76 years, 5 females, 4 males) with overall 13 lung tumors who fulfilled all inclusion and exclusion criteria were included. The inclusion criteria were: histological proven non-small-cell lung cancer (adenocarcinoma, stage IV) without any previous oncologic treatment in a palliative, non-surgical setting with planned systemic therapy. The exclusion criteria were: unwillingness to participate in the study, contraindications to magnetic resonance imaging (MRI) or inability to tolerate MRI because of high grade dyspnea or reduced general health conditions.

### Diagnosis and Treatment of Lung Cancer

In all patients, the diagnosis of lung cancer was histologically proven with transbronchial biopsy by a board-examined pulmologist. Within 14 days of diagnosis, all included patients were examined using a standardized contrast-enhanced CT and a baseline MRI examination. Therapeutic response was categorized by a radiologist (AG) with 13 years of experience in thoracic imaging using RECIST 1.1 criteria [1] by evaluating changes in the maximum tumor axial diameter on CT after two cycles of chemotherapy. Specific therapy, length of progression-free interval, and lesion size on CT before and after treatment are listed for each patient in Table 1. Note that patient 2 deceased after the second course of chemotherapy due to causes unrelated to lung cancer. At the time of death there was no tumor progression. Data for this patient was included until termination of the second course of chemotherapy.

**Table 1:** Specific therapy, length of progression-free interval, and lesion cross section size on CT before and after treatment for each patient.

Patient	Lesion	Specific therapy	Progression-freeinterval (months)	Lesion size beforetreatment (cm)	Lesion size after twocycles of chemotherapy (cm)
1	1	Cisplatin/Platinol	4	4.4	1.8
2	1	Carboplatin/Paraplatin+Gemcitabine/Gemzar	*	5.8	5.0
3	1	Erlotinib/Tarceva	21	1.9	0.7
4	1	Carboplatin/Paraplatin+Pemetrexed/Alimta	12	3.2	2.9
5	1	Cisplatin/Platinol+Gemcitabine/Gemzar	3	6.2	5.3
6	1	Cisplatin/Platinol+Pemetrexed/Alimta	14	2.1	1.7
7	1	Erlotinib/Tarceva	10	2.9	2.7
	2			2.5	2.1
8	1	Carboplatin/Paraplatin+Pemetrexed/Alimta	5	9.3	7.3
	2			1.4	0.8
9	1	Cisplatin/Platinol+Pemetrexed/Alimta	7	3.5	2.9
	2			2.8	1.6
	3			1.9	1.5

* The patient deceased prior to tumor progression due to causes unrelated to lung cancer.

### MRI Examination

MRI was performed at a maximum of 14 days before onset of treatment and repeated at one, two, and six weeks after initiation of the first course of chemotherapy. Imaging of the thorax was performed on a 1.5 T MRI scanner (Achieva, Philips Healthcare, Best, the Netherlands, Release 3.2.2.0) with the patient in the supine position using a 16-element sensitivity-encoding torso receive-only coil array (Philips Healthcare, Best, the Netherlands) covering the chest.

Axial Imaging of the thorax was performed using T_1_-weighted fast spin-echo as well as dual-echo fast gradient-echo imaging, together with axial DWI, using three b-values of 100, 600 and 800 s/mm^2^. The lower b-value was chosen to diminish perfusion effects [2]. The imaging parameters are summarized in Table 2. To minimize the effects of respiratory motion, imaging was performed using a respiratory-triggering technique with a navigator placed on the right dome of the diaphragm.

**Table 2:** Overview of MRI sequence parameters.

Sequence	Repetitiontime (ms)	Echo time(ms)	FOV (mm^2^)	Voxel size(mm^2^)	SliceThickness(mm)	No. ofslices	No. of signalaverages	Acquisition time (s)
T_1_-weighted fast SE	796	26	280×238	0.8×0.8	6	30	1	23.9
Dual-echo breathhold fast GE	5.9	2.3/4.6	375×295	1×1	6	25	1	34.8
DWI withnavigator-triggered SEecho-planar imaging andSPIR*	2174	62.1	280×233	2×2	6	30	6	400

Note: GE = gradient echo, SE = spin echo, SPIR = spectral presaturation with inversion recovery. All sequences were axial and two-dimensional.

*This sequence was performed with b-values of 100, 600, and 800 s/mm^2^ and a parallel imaging reduction factor of 1.8. The actual repetition and scan times were longer due to navigator triggering.

During the baseline scan prior to therapy, the DWI scan was acquired twice to allow for the calculation of the thresholds for the fDMs (see further details below). Therefore, the first scan session was longer than the follow-up scans. The scan times for baseline was about 20 minutes and for follow-up examinations approximately 14 minutes. Due to the respiratory-triggered DWI acquisition, the individual scan times varied between subjects.

### Diffusion Data Analysis

Data analysis was performed using in-house software written in Matlab (The Mathworks, Natick, MA, USA, Release 2010a). First, eddy current-induced image warping was corrected in the in-vivo data sets using a correlation-based affine registration algorithm [20]. Second, ADC maps were calculated using a mono-exponential fit of all b-factor images at each measurement time point. This resulted in altogether five ADC maps per patient, two pretreatment as well as three posttreatment ADC maps. Third, the second pretreatment ADC map and all posttreatment ADC maps were coregistered to the corresponding first pretreatment ADC map for each patient individually. Coregistration was performed using a robust multiresolution alignment algorithm [21] that was implemented in Matlab. The algorithm was extended to allow for affine transformations. As a quality indicator of coregistration, Pearson’s correlation coefficients of the two pretreatment ADC maps of each patient were calculated before and after coregistration. Thereby, the correlation coefficient was computed over the entire data sets.

#### Region of Interest Analysis

The lesion borders were defined manually on the ADC maps taking into account the diagnostic information of CT and the corresponding conventional anatomical MRI. The regions of interest (ROIs) were drawn over each tumor bearing slice on the ADC maps across the entire metastases by a single radiologist (AG). ROIs were defined across all lesions in each patient for each examination separately, i.e. before initiation of chemotherapy (first pretreatment ADC map) (mean size = 57.16 cm^3^; range = 1.46–253.25 cm^3^) and at one (mean size = 45.13 cm^3^; range = 1.37–247.46 cm^3^), two (mean size = 43.40 cm^3^; range = 1.30–231.36 cm^3^), and six (mean size = 33.57 cm^3^; range = 1.39–184.78 cm^3^) weeks after therapy onset. Care was taken to exclude cavitary areas or atelectatic lung regions. Thereafter, the mean ADC of each lesion at every time point was calculated and subsequently the corresponding ADC changes relative to the pretreatment values.

#### FDM Analysis

Previous work showed that, time permitting, thresholds for fDMs should be determined directly in the tumor rather than in reference tissue to maximize their accuracy [22]. Therefore, the thresholds were calculated for each patient individually directly in the tumor tissue by statistical comparison of the two pretreatment ADC maps. Thereby, the thresholds were set to the repeatability limit of the tumor tissue in each patient determined using one-way analysis of variance (ANOVA) [14]. Beyond the significance threshold, a cluster size threshold was set at six to exclude isolated voxels which most likely correspond to false-positive results [14]. In doing so, the percentage of voxels that showed a significant increase (red voxels), a significant decrease (blue voxels) or no change (green voxels) in their ADCs at each posttreatment time point relative to the pretreatment values was computed for each and every lesion in the study cohort. In this way, fDMs were generated for the intersection of each baseline ROI and the corresponding posttreatment ROI.

### Statistical Analysis

Changes in tumor diameter on CT before and after two cycles of chemotherapy were compared on a per-lesion basis using Wilcoxon signed-rank test. The mean ADC changes on a per-lesion basis and percentages of voxels with significantly increased (red voxels) and significantly decreased (blue voxels) on fDMs over time were analyzed using repeated measures ANOVA. For this analysis, the changes in tumor size on CT were first entered as covariate and second as between-subjects factor. For the latter each lesion was classified in agreement with RECIST criteria as either showing stable disease (<30% decrease in tumor size) or partial response (>30% decrease in tumor size) under anticancer treatment.

Due to the low number of patients a Kaplan-Meier survival analysis was not performed. However, to evaluate the diffusion parameters as predictive biomarkers of treatment outcome, Pearson correlation coefficients between the length of the progression-free interval and the diffusion parameters (i.e. mean ADC changes and percentages of red and blue voxels on fDMs) at the first time point after initiating treatment were calculated. In patients with multiple lesions, averaged diffusion parameters were therefore calculated on a per-patient basis as weighted means according to the size of each lesion in the patient. For this predictive analysis, data of patient 2 who died prior to tumor progression from causes unrelated to lung cancer was excluded.

Statistical analysis was performed using SPSS (IBM Corporation, Armonk, NY, USA, SPSS Statistics for Windows, Version 21.0), with p<0.05 considered to be statistically significant for each analysis.

## Results

### CT-Based Changes in Tumor Size after Two Cycles of Chemotherapy

Across the study cohort, the Wilcoxon signed-rank test showed a highly significant decrease in tumor diameter on CT (p<0.001) after two cycles of chemotherapy (mean size before therapy = 3.7 cm; range = 1.4–9.3 cm; mean size after two courses of chemotherapy = 2.8 cm; range = 0.7–7.3 cm). No patients showed an increase in tumor size after treatment.

### Diffusion Data Analysis

No violations of the assumptions of repeated measures ANOVA were observed. To this end, analysis of the residuals was performed by examining normal plots of the residuals and plots of the residuals versus the fitted values. Beyond that, Mauchly’s sphericity test was computed.

#### ROI Analysis of Mean ADC Changes During Therapy

**Figure 1:**
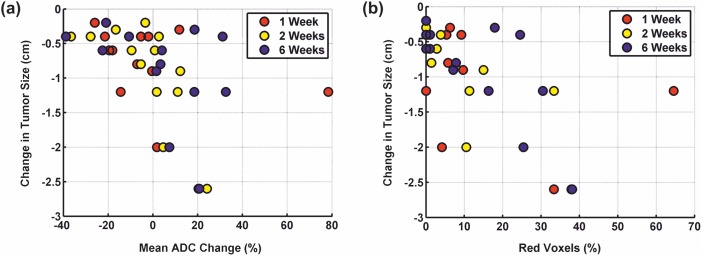
Plots of the diffusion parameters against the changes in tumor volume on CT. Plots of **(a)** the mean ADC change and **(b)** the percentage of voxels with significantly increased ADCs relative to their pretreatment values (red voxels) on fDMs in each lesion at one, two, and six weeks after initiation of chemotherapy against reduction in tumor size after two cycles of chemotherapy. Repeated measures ANOVA revealed that a large reduction in tumor size on CT was typically preceded by a large increase in the mean lesion ADC (p = 0.001) as well as a high percentage of red voxels on fDMs (p = 0.002).

The results of the ROI analysis are summarized in Table 3. Repeated measures ANOVA revealed no significant alterations in the changes of the mean tumor ADC over time (p = 0.554). However, there was a significant dependence of the mean tumor ADC change on the change in tumor size (p = 0.011). Figure 1a shows the mean ADC change of each lesion at one, two, and six weeks after initiation of chemotherapy plotted against the reduction in tumor size on CT after two cycles of chemotherapy. To understand this dependence better, changes in tumor size were recorded as a binary variable and used as a between-subjects factor for analysis. Thereby, each lesion was classified according to RECIST criteria as either showing stable disease (<30% decrease in tumor size; n = 9) or partial response (>30% decrease in tumor size; n = 4) under chemotherapy. The relationship between size reduction and mean ADC change was still significant and revealed that the mean ADC increase was larger in lesions that showed a large reduction in tumor size on CT compared with lesions that showed only a moderate decrease in tumor size after two cycles of chemotherapy (p = 0.045). In fact, lesions that demonstrated a large decrease in tumor size showed on average an increase in the mean ADCs across all measurement timepoints (mean at one week = 16.2%; mean at two weeks = 9.4%; mean at six weeks = 19.0%) whereas those with a moderate decrease in tumor size revealed a decrease in the mean ADCs across all measurement timepoints (mean at one week = −7.4%; mean at two weeks = −8.9%; mean at six weeks = −3.5%).

**Table 3:** Results of the ROI analysis of the ADCs on a per-lesion basis.

Patient	Lesion	Mean ADC beforetreatment(10^−3^ mm^2^/s)	Mean ADC change 1week after initiation ofchemotherapy(10^−3^ mm^2^/s)	Mean ADC change 2weeks after initiation ofchemotherapy (10^−3^ mm^2^/s)	Mean ADC change 6weeks after initiation ofchemotherapy (10^−3^ mm^2^/s)
1	1	1.436	0.293	0.348	0.298
2	1	1.348	−0.095	−0.073	0.045
3	1	1.007	0.789	0.111	0.328
4	1	1.341	0.158	−0.223	0.249
5	1	1.454	−0.006	0.177	0.022
6	1	1.702	−0.034	0.046	0.529
7	1	1.215	−0.316	−0.043	−0.255
	2	1.268	−0.273	−0.353	−0.136
8	1	0.998	0.018	0.046	0.074
	2	1.286	−0.251	0.010	0.052
9	1	1.823	−0.331	−0.173	−0.411
	2	1.906	−0.275	0.032	0.353
	3	1.940	−0.105	−0.71	−0.755

#### FDM Analysis of ADC Changes with Therapy

As a quality indicator of coregistration, Pearson’s correlation coefficients between the two pretreatment ADC maps were calculated before and after coregistration. The mean correlation coefficient averaged over all patients increased from 0.74 (range = 0.56–0.93) before coregistration to 0.82 (range = 0.70–0.94) after coregistration.

**Figure 2:**
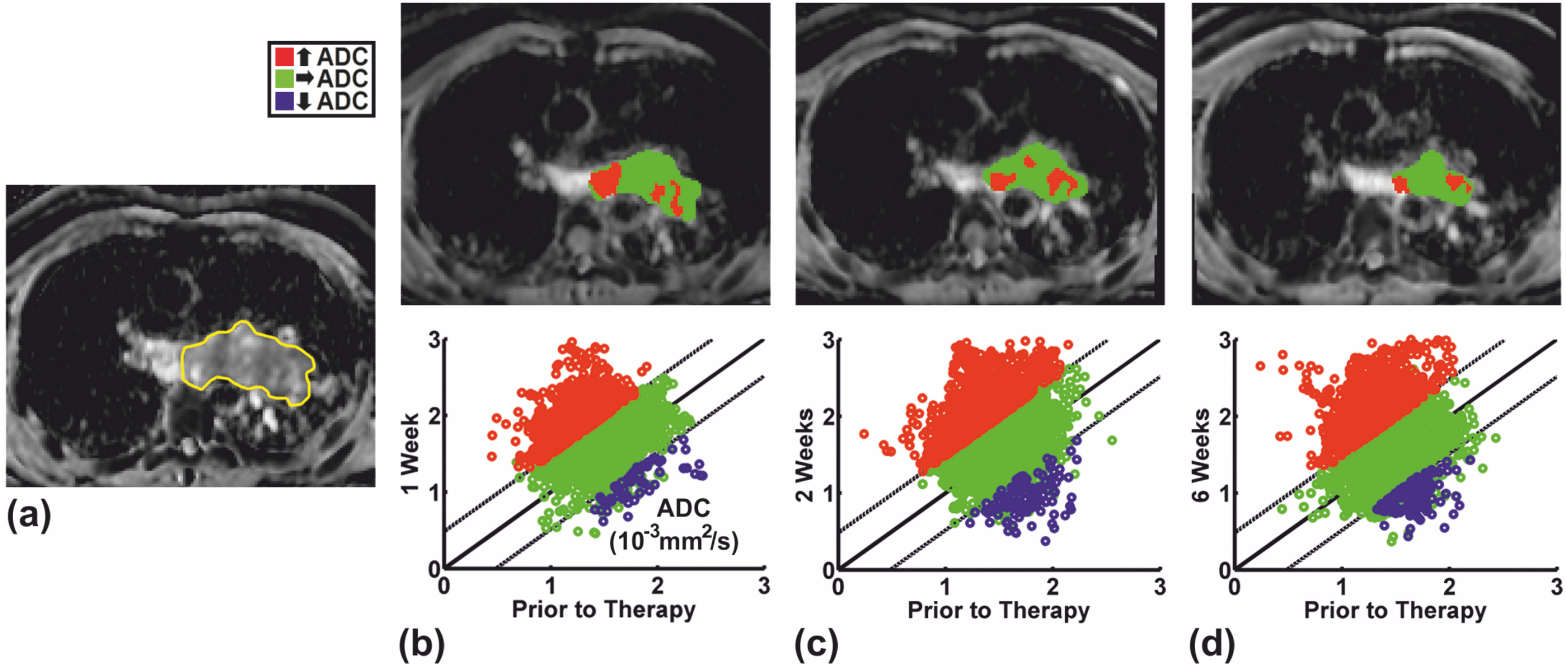
FDMs of patient 1 whose lesion showed a large reduction in tumor size under chemotherapy. **(a)** Pretreatment ADC map showing the ROI drawn by the radiologist and fDMs at, **(b)** one, **(c)** two, and, **(d)** six weeks after initiation of chemotherapy superimposed onto the corresponding posttreatment ADC map. The scatterplots of pretreatment versus posttreatment voxel values over the entire lesion are shown below each image. Dashed lines = threshold beyond which a significant ADC change is deemed to have occurred. Large regions within the lesion showed significantly increased ADCs (shown in red) at all time points after initiation of chemotherapy in comparison with pretreatment values. Note that there are voxels labeled with unchanged ADCs (green voxels) lying beyond the significance threshold on the scatterplots; these were isolated voxels that were excluded by the cluster size threshold.

**Figure 3:**
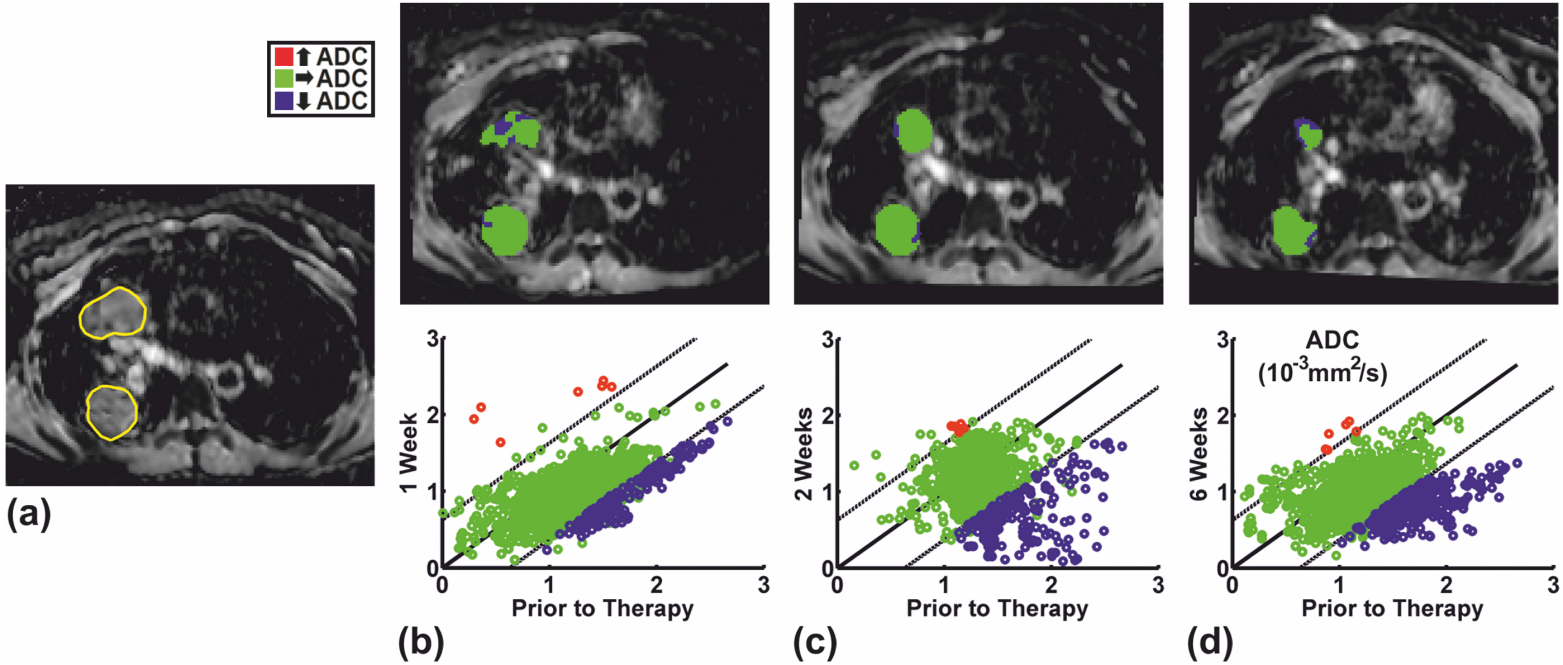
FDMs of the two lesions in patient 7 which showed a moderate decrease in tumor size under chemotherapy. **(a)** Pretreatment ADC map showing the ROIs circumscribing the tumors and fDMs at, **(b)** one, **(c)** two, and **(d)** six weeks after treatment onset superimposed onto the corresponding posttreatment ADC map. The scatterplots of pretreatment versus posttreatment voxel values over the entire lesions are shown below each image. Dashed lines = threshold beyond which a significant ADC change is deemed to have occurred. Few voxels feature significantly increased ADCs relative to the pretreatment values.

The mean threshold for the fDMs of all patients beyond which a significant ADC change was deemed to have occurred was 0.64•10^−3^ mm^2^/s (range = 0.26–0.89•10^−3^ mm^2^/s). By way of example, fDMs for two patients through a single level of the lung tumors at one, two, and six weeks after initiation of chemotherapy as well as the corresponding scatterplots over the entire lesions are depicted in Figures 2 and 3. Figure 2 depicts the fDMs of the lesion in patient 1 which showed a large reduction in tumor size on CT after two cycles of chemotherapy. Large regions with significantly increased ADCs (shown in red) are observed at all timepoints after treatment began compared with pretreatment values. By contrast, Figure 3 illustrates that only minor regions with significantly increased ADCs but larger regions with significantly decreased ADCs (depicted in blue) were observed in the two lesions of patient 7 which showed only a moderate decrease in tumor size on CT.

Repeated measures ANOVA showed no significant changes in the percentage of voxels with significantly increased ADCs (red voxels) over time (p = 0.180). However, there was a significant dependence of the percentage of red voxels on the change in tumor size (p = 0.002). The percentages of voxels with significantly increased ADCs (red voxels) relative to their pretreatment values at one, two, and six weeks after initiation of chemotherapy are plotted against the reduction in tumor size on CT after two cycles of chemotherapy in Figure 1b. As before, the change in tumor size was dichotomized as a binary variable (large vs. moderate changes in tumor size on CT) for further analysis. The percentage of red voxels was significantly higher in lesions that showed a large reduction in tumor size than in those that showed only a moderate decrease in tumor size on CT after two courses of chemotherapy (p = 0.041). Finally, repeated measures ANOVA revealed neither a significant change in the percentage of voxels with significantly decreased ADCs (blue voxels) over time (p = 0.070) nor a significant relationship between the percentage of blue voxels and the change in tumor size (p = 0.181).

**Figure 4:**
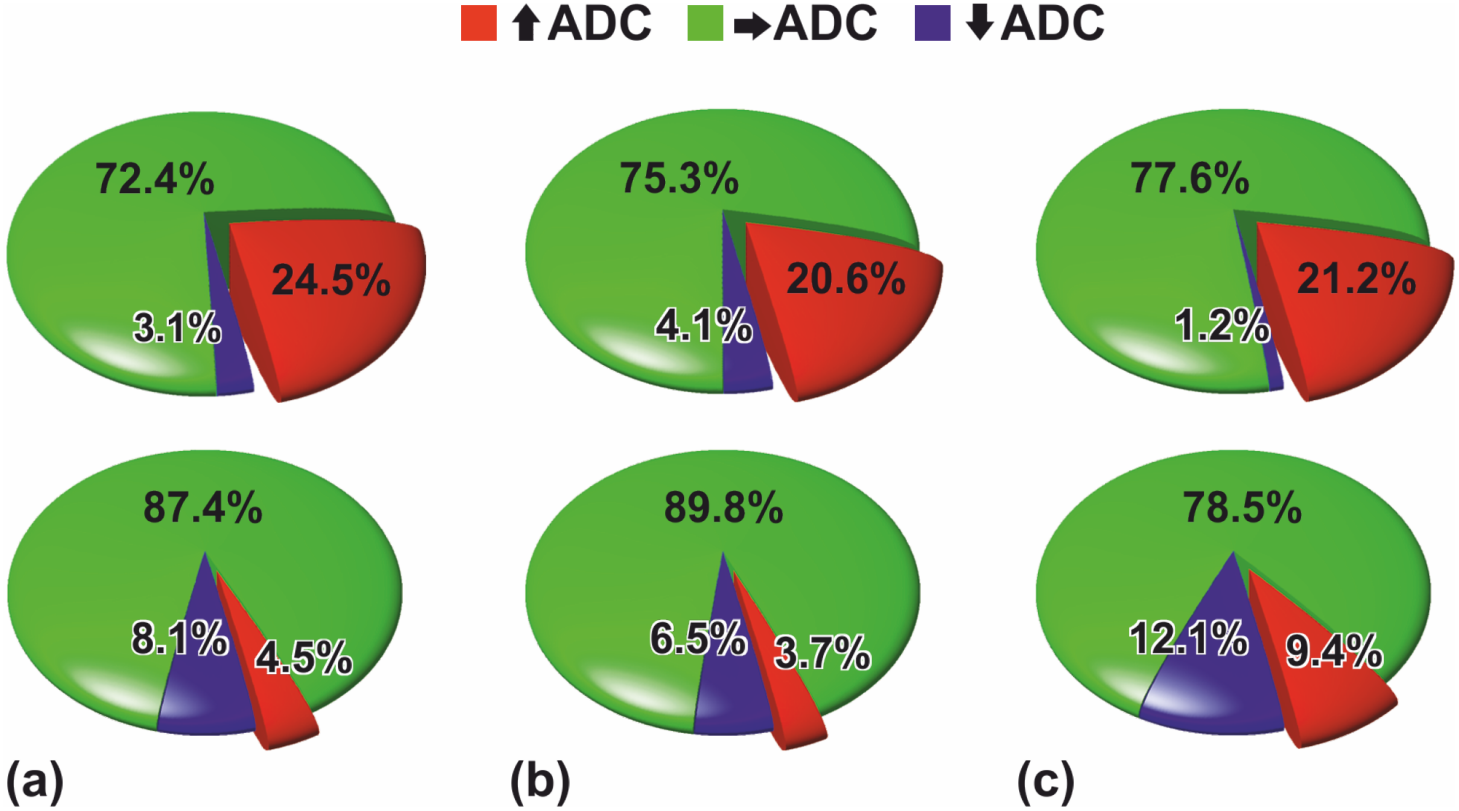
Pie charts showing the percentages of voxels on fDMs that featured significantly increased (red voxels), significantly decreased (blue voxels) or unchanged (green voxels) ADCs under therapy averaged over all lesions. The percentages at **(a)** one, **(b)** two, and **(c)** six weeks after treatment onset are depicted separately for patients that showed partial response (first row) and stable disease (second row), respectively. The percentage of voxels with significantly increased ADCs in comparison to pretreatment values was significantly higher in lesions that showed a large decrease in tumor size on CT after two cycles of chemotherapy (p = 0.041).

The pie charts in Figure 4 summarizes the results, i.e. the percentage of voxels with significantly increased ADCs compared with pretreatment values was significantly higher in lesions that showed partial response (mean at one week = 24.5%; mean at two weeks = 20.6%; mean at six weeks = 21.2%) versus lesions that only showed stable disease (mean at one week = 4.5%; mean at two weeks = 3.7%; mean at six weeks = 9.4%) on CT.

#### Pearson Correlation of Diffusion Parameters and Progression-Free Interval

Bivariate correlation did not reveal a significant relationship between the ADC change at one week after initiating treatment and the progression-free interval (r = 0.627, p = 0.096). Similarly, no significant relationships were found either between the percentages of the red voxels (r = 0.541, p = 0.166) or blue voxels (r = −0.506, p = 0.200) at one week after initiating treatment and the progression-free interval.

## Discussion

In patients diagnosed with locally advanced non-small cell lung cancer, treatment response to chemotherapy is usually assessed using RECIST 1.1 criteria [1] based on the reduction in the maximum axial tumor diameter on CT. RECIST remains the most widely accepted method for tumor response assessment even though this is made after the completion of chemotherapy, typically at 12 weeks after initiating treatment. However, as expensive targeted therapies are progressively introduced for the treatment of lung cancer [23], [24], there is a desire for early response and predictive biomarkers that would help to guide patient management.

The present preliminary study demonstrates that the percentage of voxels with significantly increased ADCs (red voxels) measured using fDMs may allow early identification of patients with partial response according to conventional RECIST criteria. FDMs may predict outcome of treatment as early as at one week after initiation of chemotherapy. For this reason, this ADC analysis method may allow physicians to make early adjustments to patient management so as to maximize treatment benefits and avoid side-effects from ineffective treatment. Furthermore, early treatment adaption could potentially render anticancer therapy more cost-efficient.

DWI of the lungs and especially longitudinal studies in lung cancer are very challenging due to respiratory motion, the presence of susceptibility-related artifacts, and the absence of rigid landmarks that simplify image coregistration. Despite these challenges, the results of our pilot study have shown that using respiratory-triggered DWI and advanced coregistration techniques, fDM analysis in pulmonary lesions is feasible and has shown promising results. This approach may prove to be more sensitive to changes resulting from therapy compared with mean ADC changes averaged over entire lesions as it accounts for heterogeneous changes that occur within each tumor with treatment. It should be noted that respiratory triggering in some patients significantly prolonged scan time due to highly irregular breathing patterns, technical improvements are required to shorten scan time in these patients while maintaining high image quality. The total processing time amounted to approximately 20 minutes per patient, limited mostly by localization and manual definition of pulmonary lesions. Automatic or semi-automatic segmentation techniques could be utilized to alleviate the time penalty and facilitate transition into clinical practice.

Unlike previous work, which relied on applying a threshold determined from a reference tissue [14] to define the level of significant voxelwise ADC change within tumors; we determined this threshold for the fDM analysis on a per-lesion basis directly in each tumor by acquiring a second ADC map prior to chemotherapy and comparing the two baseline ADC maps by one-way ANOVA. This method should improve the reliability of the fDMs [22] and may be a key factor for fDM analysis in lung cancer and other soft tissue tumors.

Our results of the mean ADC changes on a per-lesion basis are in agreement with previously published results, which have shown that an increase in the mean ADC at three to four weeks compared with pretreatment values could predict good response in patients with non-small cell lung cancer [15]. However, our study has shown that these changes could potentially be observed as early as one week after starting treatment. In addition, the information derived from fDMs, i.e. the percentage of voxels with significantly increased ADCs (red voxels) may be a more sensitive biomarker than averaged ADC changes by accounting for heterogeneity of treatment response.

It should be noted that Yabuuchi et al. [15] reported increasing mean tumor ADC values for almost all responding tumors. Intriguingly, we found that tumors that showed <30% reduction in size after two cycles of chemotherapy were on average accompanied by a decrease in their mean ADCs across all measurement timepoints. It should be mentioned at this point that contrary to Yabuuchi et al. [15] the choice of a lower b-value of 100 mm/s^2^ in our work should effectively diminish the influence of perfusion effects on ADC quantification [2]. Nevertheless, the biological basis for the observed decrease of the mean ADCs in lesions showing stable disease is uncertain. This may relate to the mechanism of cell death, associated with cellular swelling and/or inflammatory infiltrates maybe even early disease progression. Nonetheless, it would be interesting to see whether these observations could be independently validated by other investigators.

There were limitations to our study. First, a relatively small number of patients was included. The reason for this was the often poor general clinical condition of the majority of stage IV lung cancer patients, which often were not able to comply with the study requisites. Furthermore, the patient population was slightly heterogeneous with respect to specific chemotherapy administered. This might influence cellular response and in turn the diffusion properties of the lesion under therapy. This may explain why significant correlations with the progression-free interval were not found. Thereby, it should be noted that bivariate correlations had to be computed on a per-patient level, in the process further diminishing the sample size. Beyond that one patient deceased prior to tumor progression and had to be secondarily excluded from the analysis. Nevertheless, it should be noted that our results showed some trend towards statistical significance and further studies with larger and more homogeneous patient cohorts are warranted. Second, for ethical reasons, no control group without systemic treatment could be included in the present study and no patient had progressive disease. In addition, it might be interesting to investigate how the results would compare to patients treated with antiangiogenic agents in addition to conventional chemotherapy. Third, in the fDM analysis, an assumption is made that tumor regression occurs from the periphery towards the center of the tumor, allowing voxelwise registration of intersecting regions of interest before and after treatment. However, the pattern of tumor regression is likely to be more complex. Nonetheless, this approach has yielded significant results, which could be further applied and tested in future studies. In spite of these limitations, to the best of our knowledge, this is the first study demonstrating the feasibility of assessing and predicting treatment response using fDMs in patients with non-small cell lung cancer.

In conclusion, the present work demonstrates that using respiratory-triggered DWI, early treatment response can be successfully determined in patients with non-small cell lung cancer using fDMs. The percentage of voxels with significantly increased ADCs (red voxels) on fDMs may allow predicting treatment response according to RECIST criteria as early as at one week after initiation of chemotherapy. Thereby, the fDM may potentially pose a more sensitive biomarker for predicting treatment response than ADCs on a per-lesion basis by accounting for spatial heterogeneity of treatment response.
